# Draft Genome Sequence of a Hypermucoviscous Extended-Spectrum-β-Lactamase-Producing *Klebsiella quasipneumoniae* subsp. *similipneumoniae* Clinical Isolate

**DOI:** 10.1128/genomeA.00475-16

**Published:** 2016-07-07

**Authors:** U. Garza-Ramos, J. Silva-Sánchez, J. Catalán-Nájera, H. Barrios, N. Rodríguez-Medina, E. Garza-González, M. A. Cevallos, L. Lozano

**Affiliations:** a Instituto Nacional de Salud Pública (INSP), Centro de Investigación sobre Enfermedades Infecciosas (CISEI), Departamento de Diagnóstico Epidemiológico, Cuernavaca, Morelos, México; b Departamento de Medicina Interna, Hospital Universitario Dr. José Eluterio González, Nuevo León, México; c Programa de Genómica Evolutiva, Centro de Ciencias Genómicas, Universidad Nacional Autónoma de México, Cuernavaca, Morelos, México

## Abstract

A clinical isolate of extended-spectrum-β-lactamase-producing *Klebsiella quasipneumoniae* subsp. *similipneumoniae* 06-219 with hypermucoviscosity phenotypes obtained from a urine culture of an adult patient was used for whole-genome sequencing. Here, we report the draft genome sequences of this strain, consisting of 53 contigs with an ~5.6-Mb genome size and an average G+C content of 57.36%. The annotation revealed 6,622 coding DNA sequences and 77 tRNA genes.

## GENOME ANNOUNCEMENT

*Klebsiella quasipneumoniae* is a new bacterial species that has been recently described. It is broadly related to *K. pneumoniae* and *K. variicola* (1), and includes two subspecies: *K. quasipneumoniae* subsp. *quasipneumoniae* and *K. quasipneumoniae* subsp. *similipneumoniae*. Both have been exclusively isolated from human infections (2). *K. quasipneumoniae* subsp. *quasipneumoniae* and *K. variicola* with hypermucoviscosity phenotypes have been described (3, 4). A collection of 220 extended-spectrum-β-lactamase (ESBL)-producing *K. pneumoniae* clinical isolates (2005 and 2014) were retrospectively screened for hypermucoviscosity phenotype, using the semi-quantitatively string test (5). All isolates were previously identified by means of the MicrosScan Walkaway system (Dade Behring, West Sacramento, CA), and the determination of the ESBL-producing isolates was carried out by double-disc synergy test (6).

As a result of screening, the *K. pneumoniae* 06-219 isolate was identified with the hypermucoviscosity phenotype. This isolate was obtained from a urine culture of a 32-year-old man at the University Hospital in Monterrey, Nuevo Leon, Mexico in 2006. It was analyzed by multiplex-PCR (M-PCR-1) (7), showing a negative result for the molecular markers of *K. variicola*, *K. pneumoniae*, and for the *mtnC* gene that corresponds to *Klebsiella* spp. genera. Then, this isolate was used for the whole-genome sequencing.

A total genomic sample was purified using a DNeasy kit (Qiagen, Germany). The genome sequence was obtained using the Illumina (MiSeq) platform and a total of 4,705,070 pair-end reads with a length of 300-bp were obtained. Quality-based trimming was performed with the SolexaQA software and *de novo* assembly was done with SPAdes v3.1.1. In total, 57 contigs with an *N*_50_ of 1,041,587-bp were obtained. The estimated genome size was 5,645,875 bp with a 150× coverage. Gene prediction and annotation were carried out using the bioinformatic MicroScope platform (8). A total of 5,622 coding DNA sequences (CDS) and 77 tRNA genes were identified. The *rmpA* and *rmpA2* genes described in hypervirulent-*K. pneumoniae* turned out to be absent in the 06-219 genome. This bacteria contains the following virulence-associated determinants: *allABCDRS, entB*, *iroN*, *iutA*, *kfuABC*, *mrkABCDFHIJ*, *uge*, *ureA*, and *wabG*. The chromosomal OKP-B-2 and the plasmid-encoded TEM-1 and ESBL SHV-12 genes, which encode the β-lactamase genes, were identified. The operons conferring heavy metal resistance were identified: *merRTPCAPE* (mercury), *pcoABCDERS* (copper), *silCERS* (silver), *pbrACR* (lead), and *terABCDEWYZ* (tellurium). The latter has been associated with hypervirulent-*K. pneumoniae* clones (9).

To determine the bacterial specie to which isolate 06-219 belongs, an average nucleotide identity (ANI) (10) and *rpoB* gene analysis were carried out using the *K. quasipneumoniae* subsp. *quasipneumoniae* 18A069 (CBZM000000000), *K. quasipneumoniae* subsp. *similipneumoniae* 07A044 (CBZR000000000), *K. variicola* At-22 (CP001891), and *K. pneumoniae* MGH78578 (CP000647) reference genomes. A value of 99.23% for *K. quasipneumoniae* subsp. *similipneumoniae*, was obtained in comparison with a value of 96.47% obtained for *K. quasipneumoniae* subsp. *quasipneumoniae*. A value lower to 93.27% was obtained for *K. variicola* At-22 and *K. pneumoniae* MGH78578 genomes. In addition, the phylogenetic analysis of the complete *rpoB* gene showed the same results (data not shown). The above-mentioned analyses indicate that isolate 06-219 corresponds to hypermucoviscous ESBL-producing *K. quasipneumoniae* subsp. *similipneumoniae.*

### Nucleotide sequence accession numbers.

The annotated genome sequence is available at the European Nucleotide Archive under the accession numbers FKLR01000001 to FKLR01000057.
